# Early In-Bed Cycle Ergometry With Critically Ill, Mechanically Ventilated Patients: Statistical Analysis Plan for CYCLE (Critical Care Cycling to Improve Lower Extremity Strength), an International, Multicenter, Randomized Clinical Trial

**DOI:** 10.2196/54451

**Published:** 2024-10-28

**Authors:** Diane Heels-Ansdell, Laurel Kelly, Heather K O'Grady, Christopher Farley, Julie C Reid, Sue Berney, Amy M Pastva, Karen EA Burns, Frédérick D'Aragon, Margaret S Herridge, Andrew Seely, Jill Rudkowski, Bram Rochwerg, Alison Fox-Robichaud, Ian Ball, Francois Lamontagne, Erick H Duan, Jennifer Tsang, Patrick M Archambault, Avelino C Verceles, John Muscedere, Sangeeta Mehta, Shane W English, Tim Karachi, Karim Serri, Brenda Reeve, Lehana Thabane, Deborah Cook, Michelle E Kho

**Affiliations:** 1 Department of Health Research Methods, Evidence, and Impact McMaster University Hamilton, ON Canada; 2 Physiotherapy Department St. Joseph's Healthcare Hamilton Hamilton, ON Canada; 3 School of Rehabilitation Science Faculty of Health Sciences McMaster University Hamilton, ON Canada; 4 Department of Physiotherapy Austin Health Heidelberg Australia; 5 Department of Physiotherapy The University of Melbourne Parkville Australia; 6 Duke University School of Medicine Department of Orthopedic Surgery, Physical Therapy Division Durham, NC United States; 7 Li Sha King Knowledge Institute St. Michael's Hospital Toronto, ON Canada; 8 Interdepartmental Division of Critical Care University of Toronto Toronto, ON Canada; 9 Department of Medicine Faculty of Medicine and Health Sciences Université de Sherbrooke Sherbrooke, QC Canada; 10 Department of Anesthesiology Faculty of Medicine and Health Sciences Université de Sherbrooke Sherbrooke, QC Canada; 11 Centre de recherche du Centre hospitalier universitaire de Sherbrooke Sherbrooke, QC Canada; 12 Department of Medicine, Interdepartmental Division of Critical Care Medicine Toronto General Research Institute, Institute of Medical Science, University Health Network, University of Toronto Toronto, ON Canada; 13 Ottawa Hospital Research Institute University of Ottawa Ottawa, ON Canada; 14 Department of Surgery University of Ottawa Ottawa, ON Canada; 15 Department of Medicine McMaster University Hamilton, ON Canada; 16 Department of Epidemiology and Biostatistics Western University London, ON Canada; 17 Department of Medicine Western University London, ON Canada; 18 Division of Critical Care Medicine Niagara Health St. Catharines, ON Canada; 19 Department of Anesthesiology and Intensive Care Faculty of Medicine Université Laval Quebec, QC Canada; 20 Department of Family and Emergency Medicine Université Laval Québec, QC Canada; 21 Centre de recherche intégrée pour un système apprenant en santé et services sociaux Centre intégré de santé et de services sociaux de Chaudière-Appalaches Lévis, QC Canada; 22 Department of Medicine University of Maryland Medical Centre, Midtown Campus Baltimore, MD United States; 23 Division of Pulmonary and Critical Care Medicine University of Maryland School of Medicine Baltimore, MD United States; 24 Department of Critical Care Medicine Queen's University Kingston, ON Canada; 25 Department of Medicine Sinai Health System Toronto, ON Canada; 26 Department of Medicine (Critical Care) University of Ottawa Ottawa, ON Canada; 27 School of Epidemiology and Public Health University of Ottawa Ottawa, ON Canada; 28 Critical Care Division, Department of Medicine, Centre de Recherche de Hôpital du Sacré-Cœur de Montréal Hôpital Sacré-Coeur de Montréal Montreal, QC Canada; 29 Department of Medicine Brantford General Hospital Brantford, ON Canada; 30 Research Institute of St. Joseph's Hamilton, ON Canada

**Keywords:** rehabilitation, mechanical ventilation, cycle ergometry, critical illness, exercise therapy, recovery of function

## Abstract

**Background:**

Survivors of critical illness are at risk of developing physical dysfunction following intensive care unit (ICU) discharge. ICU-based rehabilitation interventions, such as early in-bed cycle ergometry, may improve patients’ short-term physical function.

**Objective:**

Before unblinding and trial database lock, we describe a prespecified statistical analysis plan (SAP) for the CYCLE (Critical Care Cycling to Improve Lower Extremity Strength) randomized controlled trial (RCT).

**Methods:**

CYCLE is a 360-patient, international, multicenter, open-label, parallel-group RCT (1:1 ratio) with blinded primary outcome assessment at 3 days post-ICU discharge. The principal investigator and statisticians of CYCLE prepared this SAP with approval from the steering committee and coinvestigators. The SAP defines the primary and secondary outcomes (including adverse events) and describes the planned primary, secondary, and subgroup analyses. The primary outcome of the CYCLE trial is the Physical Function Intensive Care Unit Test-scored (PFIT-s) at 3 days post-ICU discharge. The PFIT-s is a reliable and valid performance-based measure. We plan to use a frequentist statistical framework for all analyses. We will conduct a linear regression to evaluate the primary outcome, incorporating randomization as an independent variable and adjusting for age (≥65 years versus <65 years) and center. The regression results will be reported as mean differences in PFIT-s scores with corresponding 95% CIs and P values. We consider a 1-point difference in PFIT-s score to be clinically important. Additionally, we plan to conduct 3 subgroup analyses: age (≥65 years versus <65 years), frailty (Baseline Clinical Frailty Scale ≥5 versus <5), and sex (male versus female).

**Results:**

CYCLE was funded in 2017, and enrollment was completed in May 2023. Data analyses are complete, and the first results were submitted for publication in 2024.

**Conclusions:**

We developed and present an SAP for the CYCLE RCT and will adhere to it for all analyses. This study will add to the growing body of evidence evaluating the efficacy and safety of ICU-based rehabilitation interventions.

**Trial Registration:**

ClinicalTrials.gov NCT03471247; https://clinicaltrials.gov/ct2/show/NCT03471247 and NCT02377830; https://clinicaltrials.gov/ct2/show/NCT02377830

**International Registered Report Identifier (IRRID):**

RR1-10.2196/54451

## Introduction

### Background and Rationale

Survivors of critical illness are at risk of developing physical dysfunction that can last for 5-8 years after discharge from the intensive care unit (ICU) [1,2]. Muscle atrophy can occur rapidly during critical illness, particularly in the leg muscles, which are most vulnerable to weakness due to immobility in the ICU [3]. Quadriceps size can decrease by approximately 18% during a 10-day ICU stay, with the most significant reduction occurring within the first 3 days of admission [4]. At the 1-year follow-up, approximately 35% of ICU survivors had a below-normal 6-minute walk distance, and around 50% had not returned to work [5]. Before the pandemic, the demand for ICU services in Canada was projected to increase by 40% between 2011 and 2026. Based on a 75% survival rate [6] and a conservative estimate of a 50% post-ICU disability rate, national health care utilization costs for ICU survivors 5 years after hospitalization are estimated to exceed CAD $1.6 (US $1.18) billion [7].

Physical rehabilitation initiated in the ICU can improve patients’ functional outcomes at hospital discharge [8,9]. In a randomized trial, critically ill patients who were randomized to in-bed cycling starting 2 weeks after ICU admission had a greater 6-minute walk distance at hospital discharge compared with those receiving routine physiotherapy alone [8]. In-bed cycling initiated earlier in a patient’s ICU stay is safe [10-12] and feasible [13], but its efficacy on patients’ physical function is unknown. To address this, we planned a randomized trial comparing early in-bed cycling and usual physiotherapy versus usual physiotherapy alone. We report this statistical analysis plan (SAP) in accordance with the guidelines for the content of SAPs in clinical trials [14].

### Objectives

The primary objective of the CYCLE (Critical Care Cycling to Improve Lower Extremity Strength) randomized controlled trial (RCT) is to determine the efficacy of early in-bed cycling (initiated within 4 days of starting invasive mechanical ventilation) combined with usual physiotherapy versus usual physiotherapy alone on patients’ physical function at 3 days post-ICU discharge. We hypothesize that patients receiving in-bed cycling combined with usual physiotherapy will have better physical function at 3 days post-ICU discharge compared with those receiving usual physiotherapy alone.

## Methods

### Design

CYCLE is a 360-patient, international, multicenter, open-label, parallel-group randomized trial (1:1 ratio) with blinded primary outcome assessment at 3 days post-ICU discharge. Assessors are blinded to treatment group allocation. The study includes patients from a 46-patient internal pilot (NCT02377830).

### Ethics Approval

CYCLE is approved by the Research Ethics Boards of all participating centers and by Clinical Trials Ontario (Project 1345).

### Sites

The trial involves 17 sites across Canada, Australia, and the United States. Sites were selected through established research networks and chosen based on their interest and capacity to conduct the trial. Each site has obtained local ethics approval.

### Eligibility

The inclusion and exclusion criteria are detailed in Textbox 1.

**Trial inclusion and exclusion criteria [15].**

**1. Inclusion criteria**

- Adults (≥18 years)
- Within the first 4 days of mechanical ventilation
- Expected additional 2 days intensive care unit (ICU) stay
- Within the first 7 days of ICU admission
- Could ambulate independently before hospital admission (with or without a gait aid)

**2. Exclusion Criteria**

- Acute condition impairing patients’ ability to cycle (eg, leg fracture)
- Acute proven or suspected neuromuscular weakness affecting the legs (eg, stroke or Guillain-Barré syndrome)
- Traumatic brain injury
- Inability to follow commands in the local language before ICU admission
- Severe cognitive impairment before ICU admission
- Temporary pacemaker (internal or external)
- Pregnant (or suspected pregnancy)
- Expected hospital mortality >90%
- Body habitus unable to fit the bike (eg, leg amputation, morbid obesity)
- Specific surgical exclusion as stipulated by the surgical or ICU team
- Palliative goals of care
- Able to march on the spot at the time of screening
- Persistent therapy exemptions in the first 4 days of mechanical ventilation:
  - Increase in vasopressor/inotrope within the last 2 hours
  - Active myocardial ischemia or unstable/uncontrolled arrhythmia, as determined by the ICU team
  - Mean arterial pressure <60 mmHg or >110 mmHg, or as deemed appropriate by the treating team within the last 2 hours
  - Heart rate <40 bpm or >140 bpm within the last 2 hours
  - Persistent oxygen saturation (SpO_2_) <88% or as determined by the treating team within the last 2 hours
  - Neuromuscular blocker within the last 4 hours
  - Severe agitation (Richmond Agitation and Sedation Scale >2 [or equivalent] [16]) within the last 2 hours
  - Uncontrolled pain
  - Change in goals to palliative care
  - Team perception that in-bed cycling or physiotherapy is not appropriate for other new reasons (eg, acute peritonitis, new incision/wound, known/suspected muscle inflammation such as rhabdomyolysis)

**3. Eligible, nonrandomized exclusion criteria**

- Enrolled previously in the CYCLE (Critical Care Cycling to Improve Lower Extremity Strength) randomized controlled trial or related study
- Patient unable to give consent and no substitute decision maker (SDM) identified
- Patient or SDM declines consent
- ICU physician declines patient or SDM to be approached
- Other, specified by attending team

### Randomization

Randomization occurred after informed consent was obtained. Allocation was concealed, and a central randomization process was used. We used a web-based, comprehensive, and secure randomization service [17]. After obtaining consent, the site research coordinator logged into the website, registered the patient, and received the randomized assignment, ensuring allocation concealment. Patients were stratified by center and age (≥65 years vs <65 years).

### Intervention and Comparator

#### Intervention (Cycling + Usual Physiotherapy)

Patients randomized to cycling received 30 minutes per day of in-bed cycling, in addition to usual physiotherapy interventions, 5 days per week, during their ICU stay. Cycling continued for a maximum of 28 days or until the patient could march in place for 2 consecutive days, whichever came first.

#### Comparison (Usual Physiotherapy)

Patients randomized to usual physiotherapy received interventions according to current institutional practice. Depending on the patient’s alertness and medical stability, usual physiotherapy included activities to maintain or increase limb range of motion and strength, in- and out-of-bed mobility, ambulation, and assistance with optimizing airway clearance and respiratory function [9,18-20].

### Outcomes

#### Primary Outcome

The primary outcome for this study was the Physical Function Intensive Care Unit Test-scored (PFIT-s), measured at 3 days post-ICU discharge [21,22]. The PFIT-s includes 4 items (arm strength, leg strength, ability to stand, and step cadence), each scored from 0 to 3, summed to a maximum of 12 points, and then transformed to a total score of 10 (Table 1) [21]. Higher scores represent a better function. The PFIT-s was developed for an ICU population and includes functional items commonly performed during physical rehabilitation sessions. Unlike the 6-minute Walk Test, which is challenging to administer during ICU awakening as few patients can walk, the PFIT-s can be measured serially over time [23]. Psychometric studies of the PFIT-s identified a minimal clinically important difference of 1.0 points [21,24]. We chose the PFIT-s because we anticipated that all ICU patients could complete at least part of the assessment, even if they could not stand (eg, arm or leg strength), thereby reducing the risk of floor effects.

The PFIT-s is reliable and valid in critically ill patients, demonstrating strong psychometric properties (reliability range 0.996-1.00 [22]; convergent validity with the 6-minute Walk Test and muscle strength [21]). We selected 3 days post-ICU discharge because it is close to the intervention period and prior studies have documented variable delivery of rehabilitation post-ICU [25], which could influence later evaluations. Tables 2-4 describe the preplanned primary outcome, subgroup, and sensitivity analyses.

**Table 1:** PFIT-s^a^ scoring (adapted from Denehy et al [21]).

PFIT-s component	PFIT-s component value score				
	0	1	2	3	
Shoulder strength	MRC^b^ grade 0, 1, or 2	MRC grade 3	MRC grade 4	MRC grade 5	
Knee strength	MRC grade 0, 1, or 2	MRC grade 3	MRC grade 4	MRC grade 5	
Sit-to-stand assistance	Unable	Assist of 2 people	Assist of 1 person	No assistance	
Step cadence	Unable	>0 to 49	50 to <80	>80	

^a^PFIT-s: Physical Function Intensive Care Unit Test-scored.

^b^MRC: Medical Research Council strength grade (0-5).

**Table 2:** Description of primary outcome measure and analysis.

	Description of outcome	Measurement timing	Analysis
Physical Function Intensive Care Unit Test-scored	Based on 4 patient activities: arm strength, leg strength, ability to stand, and step cadences. Total scores range from 0 to 10 with higher scores meaning better function.	3 days after intensive care unit discharge	Linear regression, adjusted for age and clinical site

**Table 3:** Subgroup analyses.

Objective	Hypothesis	Analysis
To determine if age modifies the effect of cycling plus usual physiotherapy versus usual physiotherapy alone on the primary outcome.	Cycling will be more effective in older patients than in younger patients.	Linear regression adjusted for age and clinical site, which also includes a term for the interaction between age (≥65 years versus <65 years) and randomized allocation.
To determine if baseline clinical frailty modifies the effect of cycling plus usual physiotherapy versus usual physiotherapy alone on the primary outcome.	Cycling will be more effective in patients with baseline frailty than in patients without baseline frailty.	Linear regression adjusted for age and clinical site, which also includes the main effect of frailty (≥5 versus <5) and a term for the interaction between frailty and randomized allocation.
To determine if sex modifies the effect of cycling plus usual physiotherapy versus usual physiotherapy alone on the primary outcome.	Cycling will be more effective in male than in female patients.	Linear regression adjusted for age and clinical site, which also includes the main effect of sex and a term for the interaction between sex and randomized allocation.

**Table 4:** Sensitivity analyses.

Objective	Hypothesis	Analysis
To account for ICU^a^ mortality on the primary outcome.	Accounting for mortality will not change the effect of cycling on the primary outcome.	Linear regression, adjusted for age and clinical site. All patients will be included. Those who died before 3 days post-ICU discharge will be assigned a PFIT-s^b^ score of 0.
To determine the effect of cycling plus usual physiotherapy versus usual physiotherapy alone including only a blinded assessment of the primary outcome.	Including only patients with a blinded assessment of the primary outcome will not change the effect of cycling on the primary outcome.	Linear regression, adjusted for age and clinical site. Only patients with blinded PFIT-s assessments will be included.
To determine the effect of cycling plus usual physiotherapy versus usual physiotherapy alone under maximal protocol conditions.	Cycling will more greatly be associated with increased function in patients with higher protocol adherence.	Linear regression, adjusted for age and clinical site. Only patients who received the randomized intervention or had a temporary exemption on ≥80% of planned intervention days will be included.
To determine the effect of cycling plus usual physiotherapy versus usual physiotherapy alone in those patients with a completed assessment of the primary outcome.	Including only patients with a complete assessment of the primary outcome will not change the effect of cycling on the primary outcome.	Linear regression adjusted for age and clinical site. Only patients with a total score for the PFIT-s at 3 days post-ICU discharge will be included.
To determine if the cycling effect is affected by the center, we will conduct an analysis adjusting for age only.	Excluding adjustment for clinical sites will not change the estimated effect of cycling on the primary outcome.	Repeat the primary linear regression adjusted for age only (ie, exclude clinical site).

^a^ICU: intensive care unit.

^b^PFIT-s: Physical Function Intensive Care Unit Test-scored.

#### Secondary Outcomes

Secondary outcomes include performance-based measures, patient-reported outcomes, and those collected by chart review. Performance-based measures include muscle strength (Medical Research Council Sum Score) [26,27] and function (30-second Sit-to-Stand Test [28,29] and 2-minute Walk Test [30]). The 30-second Sit-to-Stand Test and 2-minute Walk Test are reliable in critically ill or frail older adult populations and also have age- and sex-matched norms [29,30]. Patient-reported measures included the Patient-Reported Functional Scale for ICU [31,32], critical care–related psychological distress using the Intensive Care Psychological Assessment Tool [33,34], health-related quality of life using the EuroQoL (EQ-5D-5L) [35-37], and the Hospital Anxiety and Depression Scale [38]. We also collected data on frailty (Clinical Frailty Scale) [39], Katz activities of daily living scale [40], duration of mechanical ventilation, length of stay (ICU and hospital), mortality at multiple time points (ICU, hospital, and 90-day postrandomization), and changes in living location at hospital discharge from baseline. Because of funding limitations, 90-day postrandomization outcomes were restricted to patients enrolled after March 7, 2018. Table 5 outlines our preplanned secondary outcome analyses and their timing.

**Table 5:** Description of secondary outcome measures and analyses.

Outcome	Description of outcome	Measurement timing	Analysis
Physical Function Intensive Care Unit Test [21,22]	Patients complete 4 activities: arm strength, leg strength, ability to stand, and step cadences. Total scores range from 0 to 10 with higher scores representing better function. The PFIT-s^a^ has strong psychometric properties (reliability range 0.996-1.00; convergent validity with the 6-minute walk distance and muscle strength) [21,22].	ICU^b^ awakening, ICU discharge, and hospital discharge	Includes survivors at each time point. Separate linear regressions for each time point, adjusted for age.
Medical Research Council Sum Score [41,42]	Standardized physical examination of 6 muscle groups (3 upper and 3 lower), using a 6-point scale (0=no contraction; 5=contraction sustained against maximal resistance), summed to a total score. Total scores range from 0 to 60 with higher scores representing more strength. The MRC^c^ Sum Score has excellent interrater reliability (ICC^d^ 0.98, 95% CI 0.95-1.00) [26].	ICU awakening, ICU discharge, 3 days after ICU discharge, and hospital discharge	Includes survivors at each time point. Separate linear regressions for each time point, adjusted for age.
ICU-Acquired Weakness - Medical Research Council Sum Score, categorized as <48 versus ≥48 [41,42]	Standardized physical examination of 6 muscle groups (3 upper and 3 lower), using a 6-point scale (0=no contraction; 5=contraction sustained against maximal resistance), summed to a total score. Total scores range from 0 to 60 with higher scores representing more strength.	ICU awakening, ICU discharge, 3 days after ICU discharge, and hospital discharge	Includes survivors at each time point. Separate logistic regressions for each time point, adjusted for age.
30-second Sit-to-Stand Test [28,43]	Patients complete as many full sit-to-stand repetitions as possible within 30 seconds. Higher scores represent better strength. The 30-second Sit-to-Stand Test has good interrater reliability with critically ill patients (ICC 0.85, 95% CI 0.76-0.90) [44].	ICU awakening, ICU discharge, 3 days after ICU discharge, and hospital discharge	Includes survivors at each time point. Separate linear regressions for each time point, adjusted for age.
2-minute Walk Test [30,45]	Patients walk as far as possible over 2 minutes. Higher scores represent better endurance. The 2-minute Walk Test has good interrater reliability with critically ill patients (ICC 0.78, 95% CI 0.66-0.87) [44].	ICU discharge, 3 days after ICU discharge, and hospital discharge	Includes survivors at each time point. Separate linear regressions for each time point, adjusted for age.
Intensive Care Psychological Assessment [33]	Patients answer 10 questions related to psychological distress in the ICU using a 3-point scale (0=no; 1=yes, a bit; and 2=yes, a lot), summed to a total score. Total scores range from 0 to 20, with higher scores representing more distress. The Intensive Care Psychological Assessment has good test-retest reliability (*r*=0.8) and concurrent validity with other measures of anxiety and depression [33].	Following the ICU awakening assessment	Includes survivors. Linear regression, adjusted for age.
Patient-reported functional score for ICU [46]	Patients answer 6 questions about their current perception of function, using an 11-point scale (0=unable to the perform activity; 10=able to the perform activity at the same level as before ICU admission), summed to a total score. Total scores range from 0 to 60, with higher scores representing better function. The patient-reported functional score for ICU has excellent interrater reliability (ICC 0.91, 95% CI 0.76-0.97) [46].	ICU discharge, hospital discharge, and 90 days postrandomization	Includes survivors at each time point. Separate linear regressions for each time point, adjusted for age.
Euro-QOL 5D-5L Index [37]	Patients answer 5 questions about their current perception of mobility, self-care, usual activities, pain/discomfort, and anxiety/depression, scored according to a prescribed algorithm. Higher scores represent better perceptions of health.	ICU discharge, hospital discharge, and 90 days postrandomization	Includes survivors at each time point. Separate linear regressions for each time point, adjusted for age.
Euro-QOL Visual Analogue Scale [37]	Patients rate their overall health on a 100-point visual analog scale (0=worst health; 100=best health).	ICU discharge, hospital discharge, and 90 days postrandomization	Includes survivors at each time point. Separate linear regressions for each time point, adjusted for age.
Katz Activities of Daily Living scale [40]	The patient’s ability to complete 6 tasks: bathing, dressing, toileting, feeding, continence, and bed mobility. A rater assesses whether the patient is dependent or independent according to prespecified criteria. Total scores range from 0 to 6, with higher scores representing better function.	ICU discharge and hospital discharge	Includes survivors at each time point. Separate linear regressions for each time point, adjusted for age.
Clinical Frailty Scale [39]	Frailty includes a reduction in physical reserve and loss of function across multiple body systems. The clinical frailty scale is a 9-point scale, with higher scores representing more frailty. The Clinical Frailty Scale is reliable by chart review conducted by ICU research coordinators, occupational therapists, and geriatric residents [47].	Hospital discharge and 90 days postrandomization	Includes survivors at each time point. Separate linear regressions for each time point, adjusted for age.
Hospital Anxiety and Depression Scale [38]	Patients answer 14 questions on a 4-point scale (7 related to anxiety and 7 related to depression); higher scores (maximum 21 points) represent worse anxiety or depression.	90 days postrandomization	Includes survivors. Linear regression, adjusted for age.
Duration of mechanical ventilation	Days of invasive mechanical ventilation via endotracheal tube or tracheostomy	ICU discharge	Linear regression, adjusted for age
ICU length of stay	Days in ICU	ICU discharge	Linear regression, adjusted for age
Hospital length of stay	Days in hospital	Hospital discharge	Linear regression, adjusted for age
Mortality	Death	ICU discharge, hospital discharge, and 90 days postrandomization	Separate Cox proportional hazards models for each time point, adjusted for age
Hospital discharge location	Same or better living location at hospital discharge from baseline	Hospital discharge	Includes survivors. Logistic regression, adjusted for age

^a^ICU: intensive care unit.

^b^MRC: Medical Research Council.

^c^PFIT-s: Physical Function Intensive Care Unit Test-scored.

^d^ICC: intraclass coefficient.

#### Adverse Events

We collected data on the following adverse events if they occurred during or immediately after in-bed cycling or usual physiotherapy interventions, were attributed by the clinical team to the randomized intervention, and resulted in clinical deterioration of the patient’s status [8,12,48-50]: concern for myocardial ischemia or suspected new unstable/uncontrolled arrhythmia; sustained symptomatic bradycardia (<40 bpm) or tachycardia (>140 bpm); sustained hypertension (mean arterial pressure >120 mmHg); sustained oxygen desaturation below baseline (typically <90% or 88%); marked ventilator dyssynchrony; bleeding at the femoral catheter site; and new bruising at the femoral catheter site. Serious adverse events included unplanned extubation, cardiac arrest, or falls to the knees during usual physiotherapy activities.

### Sample Size

Our sample size of 360 patients was determined to detect a 1.0-point mean difference [51] between the cycling + usual physiotherapy group and the usual physiotherapy group for the PFIT-s measured at 3 days after ICU discharge [21,22]. Previous psychometric studies of the PFIT-s identified the minimal clinically important difference as 1.0 point [21,24]. Logistic regression analysis of patients enrolled in the TryCYCLE [12] and the CYCLE pilot randomized study [13] found that each 1.0-point increase in PFIT-s at ICU discharge (indicating better function) was associated with a 40% reduction in the composite outcome of death, readmission to ICU, or requiring paid assistance for activities of daily living at hospital discharge [51]. Based on an SD of 2.5 points at ICU discharge [12,52], a 1.0-point difference between groups [21,24,51], and 90% power (α=.05), we estimated the need to randomize and analyze 266 patients (133 per group). Based on data from 66 patients enrolled in the CYCLE Pilot RCT, we anticipated approximately 35% total attrition (25% ICU mortality, 1% mortality in the first 3 days post-ICU discharge, 5% missed primary outcome assessments at 3 days post-ICU, and 5% unblinded). Therefore, we recruited a total of 360 patients.

### Framework

This trial was designed as a superiority trial, hypothesizing that patients receiving in-bed cycling combined with usual physiotherapy early in their ICU stay will have better physical function at 3 days post-ICU discharge compared with those receiving usual physiotherapy alone.

### Statistical Analysis

#### Interim Analysis

We conducted a blinded interim analysis of the first 180 patients enrolled (half of the sample size) to assess benefits and harms, including serious adverse events. We adhered to conservative statistical guidelines for data monitoring based on the modified Haybittle-Peto rule [53]. The Data Monitoring Committee recommended continuing the trial on September 29, 2020, based on this interim analysis. To maintain the overall type-I error rate (ie, α), we evaluated the primary endpoint using a fixed conservative α of .001 for the interim analyses and plan to use α of .05 for the final analysis.

#### Timing of Final Analysis

The first publication of the trial results will focus on comparing the cycling + usual physiotherapy group with the usual physiotherapy group once every patient has reached 90 days postrandomization and data on vital status at hospital discharge have been received. Longer-term endpoints for the economic evaluation will be reported in a separate publication. This document will outline only the analyses included in the primary CYCLE manuscript.

#### Timing of Outcome Assessments

Table S1 in Multimedia Appendix 1 outlines the schedule of study procedures with 5 time points for outcome assessments.The ICU awakening time point was based on the physiotherapist’s assessment of the patient's ability to consistently follow 3 out of 5 verbal commands [42]. A patient’s discharge might be delayed for reasons unrelated to their readiness for discharge (eg, unavailability of hospital beds in the transfer ward). Therefore, ICU discharge measures were recorded either when the patient was discharged from the ICU or when a discharge order was written, whichever occurred first. The 3-day post-ICU time point was scheduled for 3 days after the patient’s physical discharge from the ICU. The hospital discharge time point was recorded when a discharge order was written for the patient for the index admission, including transfer to an alternative level of care. The 90-day time point was scheduled for 90 days after randomization.

#### Other Principles

All statistical tests will be 2-sided and performed at a 5% significance level. We will report 2-sided 95% CIs and conduct all analyses using SAS version 9.4. Our final RCT report will adhere to (1) the CONSORT (Consolidated Standards of Reporting Trials) 2010 Statement for reporting parallel group randomized trials (Multimedia Appendix 2) [54]; (2) the Extension for Reporting Trials of Nonpharmacologic Treatments [55]; (3) the Guidelines for Reporting Outcomes in Trial Reports, The CONSORT-Outcomes 2022 Extension [56]; and (4) the CONSORT 2024 Statement [57].

#### Trial Profile

**Figure 1:**
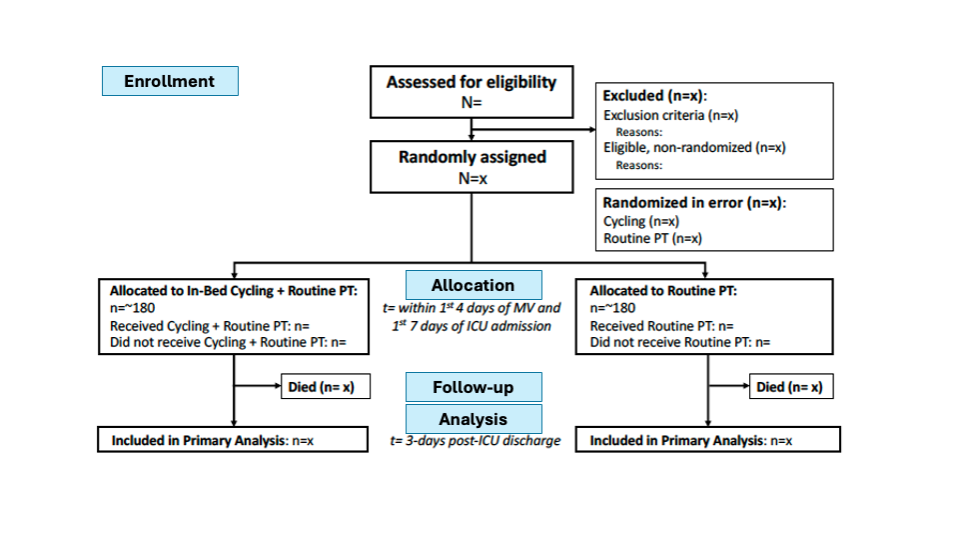
CONSORT (Consolidated Standards of Reporting Trials) patient flow diagram.

We will report the total number of patients screened, including those meeting all inclusion criteria and those with exclusion criteria, based on screening logs from participating sites. For eligible patients, we will provide reasons for non-enrollment. Patient withdrawals and losses to follow-up will be documented in our CONSORT diagram (Figure 1).

#### Protocol Adherence

The following definitions are used in this study.

“Study days” included all days in the ICU from the day of randomization to 28 days postrandomization.

We did not plan for the randomized intervention to occur under the following circumstances:

- on days when a patient was randomized after normal physiotherapist working hours;
- on days when a patient was transferred out of the ICU before 12:00 pm;
- on weekends or statutory holidays;
- for patients randomized to in-bed cycling, if they had marched on the spot for 2 consecutive days and continued marching or had higher mobility for the remainder of their ICU stay; and
- on days when a patient did not meet the criteria to receive usual physiotherapy, based on institutional policies for the delivery of usual care.

The remaining days were designated as “Planned intervention days.” On weekdays (ie, nonholiday Monday through Friday), physiotherapists reviewed study patients for 1 or more of the following “Temporary exemptions” before offering the randomized intervention:

- increase in vasopressor/inotrope within the last 2 hours;
- active myocardial ischemia or unstable/uncontrolled arrhythmia, as determined by the ICU team;
- mean arterial pressure <60 mmHg or >110 mmHg, or as deemed appropriate by the treating team within the last 2 hours;
- heart rate <40 bpm or >140 bpm within the last 2 hours;
- persistent oxygen saturation (SpO_2_ <88% or as determined by the treating team within the last 2 hours;
- receipt of neuromuscular blocker within the last 4 hours;
- severe agitation (Richmond Agitation and Sedation Scale >2 [or equivalent] [16]) within the last 2 hours;
- uncontrolled pain;
- change in goals to palliative care; and
- team perception that in-bed cycling or therapy was not appropriate for other new reasons (eg, acute peritonitis, new incision/wound, known/suspected rhabdomyolysis).

If the patient had no “temporary exemptions,” we proceeded with offering the randomized intervention.

Each “planned intervention day” without a “temporary exemption” was considered an “eligible day.” An eligible day where the patient did not receive the randomized intervention was classified as a “missed opportunity.” Missed opportunities may have occurred due to the following:

- patient factors (eg, patient unavailable due to a test or declined intervention);
- therapist factors (eg, therapist unavailable due to vacation or illness); and
- equipment factors for patients in the cycling arm (eg, malfunction of the cycle ergometer).

We define percent adherence as the ratio of days on which patients received the randomized intervention or had a temporary exemption (numerator) to all planned intervention days, including days with the randomized intervention, temporary exemptions, and missed opportunities (denominator). Descriptive statistics on percent protocol fidelity will be reported for the cohort, broken down by randomization group.

#### Major Protocol Deviation

If a patient randomized to usual physiotherapy alone received cycling, this was considered a major protocol deviation.

### Analysis Populations

#### Patient Inclusion and Outcome Analysis

We will include all eligible randomized patients (ie, excluding postrandomization exclusions representing noneligible patients) according to the treatment they were randomized to receive. Analyses of the primary outcome will be restricted to patients who survived to 3 days post-ICU discharge, as specified in our original protocol and sample size calculation [15]. Analyses of the PFIT-s at other time points (ICU awakening, ICU discharge, and hospital discharge) and all performance-based (strength and function) and patient-reported (eg, quality of life) outcomes will be restricted to patients who survived to the respective time point. We will include only patients discharged alive from the hospital in the analysis of hospital discharge location. The analyses of the duration of mechanical ventilation, ICU and hospital length of stay, and mortality will include all enrolled patients.

#### Analysis of the Primary Outcome

To determine if there is a difference in PFIT-s score at 3 days after ICU discharge between the cycling + usual physiotherapy and usual physiotherapy groups, we will conduct a linear regression analysis with randomization group (cycling + usual physiotherapy vs usual physiotherapy) as the independent variable [58]. We will adjust for age (≥65 years vs <65 years) and clinical site, as these were used as randomization stratification variables. We will report the results of the regression analysis as the mean difference in PFIT-s scores with corresponding 95% CIs and *P* values. Although the goal was to have all outcome assessors remain blinded to treatment allocation, this was not always feasible. To maximize the use of available data, we will include all PFIT-s measures at 3 days postrandomization, regardless of the blinding status of the outcome assessor, and will report the proportion of assessments conducted by blinded assessors.

To account for incomplete component data in the PFIT-s at 3 days post-ICU, we will concurrently consider data from the PFIT-s, 30-second Sit-to-Stand, and 2-minute Walk tests. We will evaluate all PFIT-s data components at 3 days post-ICU discharge. We will identify patients with any incomplete physical function data and review the scores for all 4 components of the PFIT-s (ie, shoulder flexion, knee extension, level of assistance required for sit-to-stand, and step cadence). In the PFIT-s, a score of “0” indicates a lack of physical ability to complete the measure. Therefore, if a patient attempts an item and is unsuccessful, the item receives a score of “0”, which accurately reflects their performance (Tables 1). Further details are provided in Tables S2 and S3 in Multimedia Appendix 1. Table 2 describes the primary outcome analysis.

#### Subgroup Analyses

We will conduct 3 exploratory a priori subgroup analyses to investigate potential treatment effect modification for the primary outcome.

- age ≥65 years versus <65 years;
- baseline clinical frailty ≥5 versus <5; and
- male versus female.

In separate linear regression models for each of the 3 subgroup analyses, we will include randomized treatment allocation, the subgroup variable, and the interaction between the subgroup variable and randomized treatment allocation as independent variables. These analyses will be adjusted for age and center. We hypothesize that the treatment effect will be greater for older patients compared with younger patients [59], greater in patients with frailty compared with those without [59], and greater in males compared with females [60,61]. For statistical significance in the subgroup analyses, we will use an α of .10 for the interaction term. We will assess the credibility of any statistically significant subgroup effect using the method described by Schandelmaier et al [62]. These data will be reported in a forest plot.

#### Sensitivity Analyses

To assess the robustness of the findings, we will conduct 5 sensitivity analyses for the primary outcome. All sensitivity analyses will be adjusted for age and center, unless specified:

- To account for ICU mortality on the primary outcome, we will include all patients who died before 3 days post-ICU discharge, assigning a PFIT-s score of 0 for these patients.
- We will conduct a linear regression analysis that includes only PFIT-s assessments performed by assessors blinded to treatment allocation.
- We will analyze data from patients who adhered to the protocol on ≥80% of planned ICU days. Adherence is defined as either receiving the randomized intervention or having a temporary exemption.
- We will investigate the effect of missing data by conducting a complete case analysis, including only patients with a total PFIT-s score at 3 days post-ICU discharge.
- To determine if the cycling effect is influenced by the center, we will conduct an analysis adjusting for age only.

See Table 4 for further details.

#### Analyses of Secondary Outcomes

For each continuous secondary outcome, we will conduct a linear regression analysis [58]. We will conduct secondary outcome analyses adjusting for age (≥65 years versus <65 years) only. To avoid the risk of overfitting, we will not adjust for center when analyzing our secondary outcomes. We will report the results of the linear regressions as mean differences with corresponding 95% CIs. If needed to normalize the data, we will perform the linear regression on the log-transformed outcome [58]. If the data are still skewed, we will perform nonparametric analyses. As secondary analyses are underpowered and therefore hypothesis-generating, we will not present *P* values. In Multimedia Appendix 1, we describe the scoring algorithm to account for incomplete data in the 30-second Sit-to-Stand and 2-minute Walk tests based on a patient’s observed function.

We will analyze time to ICU, hospital, and 90-day mortality using Cox proportional hazards regression analysis [63]. We will report hazard ratios and corresponding 95% CIs [63]. All other binary outcomes will be analyzed using logistic regression analysis, reporting odds ratios with corresponding 95% CIs [58]. We will check the assumptions of the different regression analyses by examining residuals and using other relevant methods. Table 5 describes secondary outcomes analyses.

#### Adverse Events

For the safety analysis, we will only include the days on which the patients received the randomized intervention (ie, days at risk of a safety event associated with rehabilitation activities). We will report the frequency and percentage of patients with severe and serious adverse events, by group. We will also report the frequency and percentage of randomized intervention days with severe and serious adverse events, by group.

#### Missing Data

We will use multiple imputations to account for missing data in performance-based and patient-reported outcomes [64-66]. In the Cox proportional hazards analyses for ICU and hospital mortality outcomes, we will censor patients with incomplete follow-up at the time of last contact.

#### Tables and Figures

We will summarize categorical data as counts and percentages. We will summarize continuous data as means, SDs, or median and IQR, if data are nonnormally distributed. For baseline variables, we will not conduct tests of statistical significance between randomized groups; rather, we will note the clinical importance of any imbalance between groups. We will report subgroup analyses in a forest plot.

### Document History

Version 1.0 of the SAP was finalized on January 9, 2024. It was uploaded to clinicaltrials.gov on January 24, 2024.

## Results

CYCLE was funded in 2017, and enrollment was completed in May 2023. Data analyses are complete, and the first results were submitted for publication in 2024.

## Discussion

### SAP-Specific Strengths and Limitations

The CYCLE RCT is the largest trial of in-bed cycling for critically ill, mechanically ventilated adults, to date. Strengths and limitations related to the design of the CYCLE RCT and the intervention of in-bed cycling have been discussed previously in the published protocol [15]. Briefly, we describe the strengths and limitations specific to this SAP.

A key strength of this SAP is the use of published guidelines to guide our reporting [14]. In addition, we selected outcome measures for the CYCLE RCT that have been validated in the ICU population and have strong psychometric properties. For our primary outcome, the PFIT-s, patients can complete parts of the outcome even if they are deconditioned (ie, cannot stand), limiting floor effects and maximizing the number of outcome assessments. We conducted a preplanned interim analysis once we reached half of our enrollment target, preventing the continuation of the trial with identified harm or benefit.

Our SAP also has important limitations. Given the number and types of outcome measures, we anticipate missing or incomplete data or both. Therefore, in this SAP we have specified the use of multiple imputations to account for these missing data. In addition, given the number of secondary outcomes, multiplicity is a concern. As a result, any significant findings from secondary outcomes will be exploratory rather than confirmatory.

This SAP complements the protocol paper [15] and was publicly available before data analysis (NCT03471247). We will adhere to it for all analyses, enhancing the rigor of our trial. The CYCLE RCT will add to the growing body of evidence evaluating the efficacy and safety of ICU-based rehabilitation interventions.

## Appendix

Multimedia Appendix 1 Summary assessments and scoring algorithm.

Multimedia Appendix 2 CONSORT (Consolidated Standards of Reporting Trials) checklist.

Multimedia Appendix 3 Peer Review Report - Canadian Institutes of Health Research.

## Abbreviations

CONSORT: Consolidated Standards of Reporting Trials
CYCLE: Critical Care Cycling to Improve Lower Extremity Strength
ICU: intensive care unit
MRC: Medical Research Council
PFIT-s: Physical Function Intensive Care Unit Test-scored
RCT: randomized controlled trial
SAP: statistical analysis plan
SDM: substitute decision maker
SpO2: oxygen saturation

## Data Availability

The data sets generated during or analyzed during this study are not publicly available because we did not receive participant consent for open sharing. Data will be available from the corresponding author on reasonable request 1 year after publication of the main manuscript.
