# Supplementary material for: Early In-Bed Cycle Ergometry With Critically Ill, Mechanically Ventilated Patients: Statistical Analysis Plan for CYCLE (Critical Care Cycling to Improve Lower Extremity Strength), an International, Multicenter, Randomized Clinical Trial
Source: JMIR Res Protoc. 2024 Oct 28;13:e54451. doi: 10.2196/54451 (PMC11555464; doi:10.2196/54451)
Supplement: Multimedia Appendix 1 [file resprot_v13i1e54451_app1.pdf]

Early in-bed cycle ergometry with critically ill, mechanically ventilated patients - Statistical Analysis Plan for CYCLE (Critical care cycling to improve lower extremity strength), an international, multi-centre, randomized clinical trial

Diane Heels-Ansdell[1], Laurel Kelly[2], Heather K. O'Grady[3], Christopher Farley[3], Julie C. Reid[3], Sue Berney[4,5], Amy M. Pastva[6], Karen E. A. Burns[7,8], Frédérick D'Aragon[9,10], Margaret S. Herridge[11], Andrew J. E. Seely[12,13], Jill C. Rudkowski[14], Bram Rochwerg[14,1], Alison E. Fox-Robichaud[14,15], Ian Ball[16,17], Francois Lamontagne[18,19], Erick H. Duan[14], Jennifer L. Tsang[20,14], Patrick M. Archambault[21,22], Avelino C. Verceles[23], John Muscedere[24,25], Sangeeta Mehta[26,27], Shane W. English[28,29], Tim Karachi[14], Karim Serri[30], Brenda K. Reeve[31], Lehana Thabane[1,32], Deborah J. Cook[33,34], Michelle E. Kho[3,32†]

Supplementary Material

Supplement Table 1: Summary of Assessments

	Study Period							
	**Enrolment**	**Allocation**	**Post-allocation**					
**Timepoint**	ICU admission	0	In ICU	ICU awakening	ICU D/C	3 days post-ICU D/C	Hospital D/C	90 days post-randomization
**Enrolment:**								
Eligibility screening	X							
Informed consent	X							
Allocation		X						
**Interventions:**								
In-bed cycling + Usual physiotherapy			X	X	X			
Usual physiotherapy			X	X	X			
**Assessments:**								
Severity of illness: APACHE II [1]	X							
Charlson Comorbidity Index[2]	X							
Functional Comorbidity Index[3]	X							
Clinical Frailty Scale [4]	X						X	X
Function: Katz Activities of Daily Living scale[5]	X				X		X	
Patient-reported functional scale for the ICU [6]					X		X	X
Physical Strength and Function:				X	X	X (blinded)	X (blinded)	
Physical Function ICU Test (scored)[7, 8]				X	X	X (blinded, Primary)	X (blinded)	
Medical Research Council Sum Score[9]				X	X	X (blinded)	X (blinded)	
30-second sit to stand[10]				X	X	X (blinded)	X (blinded)	
2-minute walk test[11]					X	X (blinded)	X (blinded)	
Psychological distress: Intensive Care Psychological				X				

	Study Period							
	**Enrolment**	**Allocation**	**Post-allocation**					
**Timepoint**	ICU admission	0	In ICU	ICU awakening	ICU D/C	3 days post-ICU D/C	Hospital D/C	90 days post-randomization
Assessment Tool [12]								
Health-related Quality of Life: Euro-QOL 5D-5L[13, 14]					X		X	X
Hospital Anxiety and Depression Scale [15]								X
**Data Collection:**								
Baseline demographics	X							
Co-interventions			Document daily on CRFs					
Study-related Serious Adverse and Adverse Events			Document daily on CRFs					
Duration of mechanical ventilation								
ICU and Hospital Length of Stay							X	
Mortality					X		X	X

Supplement 2: Scoring algorithm for incomplete data in the PFIT-s

To account for incomplete component data in the PFIT-s, we will concurrently consider data from the PFIT-s, 30s sit-to-stand, and 2-minute walk tests at 3 days post-ICU or relevant time point (i.e., ICU awakening, ICU discharge, hospital discharge). For each patient with incomplete PFIT-s component data, we will evaluate the item within the context of the other scored PFIT-s items. For example, if a patient had a knee strength PFIT-s score of 1 or less (reflecting a Medical Research Council score of 3 or less and representing an inability to complete movement against gravity with any resistance), and had incomplete data for the sit-to-stand assistance component, then we would code sit to stand a score of 0, as this patient would likely not have the strength to stand. Likewise, if a patient completed the sit-to-stand component, needed assistance from 2 or more people, and data for step cadence were incomplete, we would assign step cadence a score of 0, as this patient would likely not have the strength to take any steps. In contrast, if a patient required 1 person to stand, and data for step cadence were incomplete, we would code this as missing data, as we do not have enough information to assess this patient's ability to perform the assessment.

To reduce bias, two research personnel will be blinded to the patient's randomized allocation and clinical site. The research personnel will only evaluate patients with incomplete data. We will document the rationale for all decisions in the study analytic data set. The two research personnel will aim for consensus, however if there is disagreement, we will invite a third clinical research coordinator to make a final judgement. We will use the following scoring algorithm based on a patient's observed function (Table S2):

Table S2: PFIT-s scoring algorithm

		**PFIT-s Component value scored if incomplete**			
	Observed	Shoulder strength	Knee strength	Sit-to-Stand Assistance	Step Cadence
**PFIT-s Components:**					
Shoulder strength	Any	N/A	Missing	Missing	Missing
Knee Strength	0 (MRC grade 0, 1, or 2)	Missing	N/A	**0 (unable)**	**0 (unable)**
	1 (MRC grade 3)	Missing	N/A	**0 (unable)**	**0 (unable)**
	2 (MRC grade 4)	Missing	N/A	Missing	Missing
	3 (MRC grade 5)	Missing	N/A	Missing	Missing
Sit-to-Stand assistance	0 (unable)	Missing	Missing	N/A	**0 (unable)**
	1 (assist of 2 people)	Missing	Missing	N/A	**0 (unable)**
	2 (assist of 1 person)	Missing	Missing	N/A	Missing
	3 (no assistance)	Missing	Missing	N/A	Missing
Step Cadence	Unable	Missing	Missing	Missing	N/A
	Able to clear their foot more than once	Missing	Missing	Missing	N/A
**Other Performance-based outcomes:**					
30s Sit to Stand	1 or more repetitions; assistance of 2 or more people	Missing	Missing	**1 (assist of 2 people)**	**0 (unable)**
	Any score >0 m	Missing	Missing	Missing	Missing

		PFIT-s Component value scored if incomplete			
	Observed	Shoulder strength	Knee strength	Sit-to-Stand Assistance	Step Cadence
2-minute walk	Any score >0 m and information re: assistance	Missing	Missing	Missing	Missing

N/A = not applicable; MRC = Medical Research Council strength grade (0 to 5)

Supplement 3: Scoring algorithm for incomplete data in the 30 Second Sit to Stand and 2-minute walk tests

30 Second Sit to Stand (30STS): We will review the 30STS within the context of the knee strength and sit-to-stand assistance components of the PFIT-s at each corresponding time point. If a patient had a knee strength PFIT-s score of 1 or less (reflecting a Medical Research Council score of 3 or less and representing an inability to complete movement against gravity with any resistance) and had missing data for 30STS, then we would code a score of 0 repetitions, as this patient would likely not have the strength the stand. If a patient received a sit-to-stand PFIT-s item score of 0 (representing inability to stand even with assistance), we will code the 30STS a score of 0, representing 0 repetitions since this patient cannot stand.

2-minute walk test (2MWT): We will review the 2MWT within the context of the knee strength, sit-to-stand assistance, and step cadence components of the PFIT-s at each corresponding time point. If a patient had a knee strength PFIT-s score of 1 or less, then we would code the 2MWT a score of 0 m, as they likely do not have the strength to stand or walk. If a patient completed sit-to-stand and required assistance from 2 or more people, and data for 2MWT was missing, we would code the 2MWT a score of 0, as this patient would likely not have the strength to walk. If a patient received a PFIT-s step cadence score of 1 or higher (representing ability to take at least 1 step) and data for the 2MWT was missing, then we would code this as missing data, as the patient may have been able to walk some distance.

We will use the same procedures to reduce bias, as described in Supplement 2 for the PFIT-s. We will use the following scoring algorithm based on a patient's observed function (Table S3):

Table S3: 30s Sit to Stand and 2-minute walk scoring algorithm

		**Score if incomplete**	
**PFIT-s Component**	Observed	30s Sit to Stand repetitions	2-minute walk distance
Shoulder strength	Any	Missing	Missing
Knee Strength	0 (MRC grade 0, 1, or 2)	**0**	**0**
	1 (MRC grade 3)	**0**	**0**
	2 (MRC grade 4)	Missing	Missing
	3 (MRC grade 5)	Missing	Missing
Sit-to-Stand assistance	0 (unable)	**0**	**0**
	1 (assist of 2 people)	**1**	**0**
	2 (assist of 1 person)	Missing	Missing
	3 (no assistance)	Missing	Missing
Step cadence	0 (unable)	Missing	**0**
	Able to clear their foot more than once	Missing	Missing

Legend: MRC = Medical Research Council Score

References

1. Knaus WA, Draper EA, Wagner DP, Zimmerman JE: **APACHE II: a severity of disease classification system**. *Crit Care Med* 1985, **13**(10):818-829.
2. Charlson ME, Pompei P, Ales KL, MacKenzie CR: **A new method of classifying prognostic comorbidity in longitudinal studies: development and validation**. *J Chronic Dis* 1987, **40**(5):373-383.
3. Groll DL, To T, Bombardier C, Wright JG: **The development of a comorbidity index with physical function as the outcome**. *J Clin Epidemiol* 2005, **58**(6):595-602.
4. Bagshaw SM, Stelfox HT, McDermid RC, Rolfson DB, Tsuyuki RT, Baig N, Artiuch B, Ibrahim Q, Stollery DE, Rokosh E, Majumdar SR: **Association between frailty and short- and long-term outcomes among critically ill patients: a multicentre prospective cohort study**. *CMAJ* 2014, **186**(2):E95-102.
5. Katz S, Ford AB, Moskowitz RW, Jackson BA, Jaffe MW: **Studies of Illness in the Aged. The Index of Adl: A Standardized Measure of Biological and Psychosocial Function**. *JAMA* 1963, **185**:914-919.
6. Reid JC, Clarke F, Cook DJ, Molloy A, Rudkowski JC, Stratford P, Kho ME: **Feasibility, Reliability, Responsiveness, and Validity of the Patient-Reported Functional Scale for the Intensive Care Unit: A Pilot Study**. *J Intensive Care Med* 2019:885066618824534.
7. Denehy L, de Morton NA, Skinner EH, Edbrooke L, Haines K, Warrillow S, Berney S: **A physical function test for use in the intensive care unit: validity, responsiveness, and predictive utility of the physical function ICU test (scored)**. *Phys Ther* 2013, **93**(12):1636-1645.
8. Skinner EH, Berney S, Warrillow S, Denehy L: **Development of a physical function outcome measure (PFIT) and a pilot exercise training protocol for use in intensive care**. *Crit Care Resusc* 2009, **11**(2):110-115.
9. Kleyweg RP, van der Meche FG, Schmitz PI: **Interobserver agreement in the assessment of muscle strength and functional abilities in Guillain-Barre syndrome**. *Muscle Nerve* 1991, **14**(11):1103-1109.
10. Jones CJ, Rikli RE, Beam WC: **A 30-s chair-stand test as a measure of lower body strength in community-residing older adults**. *Res Q Exerc Sport* 1999, **70**(2):113-119.
11. Pin TW: **Psychometric properties of 2-minute walk test: a systematic review**. *Arch Phys Med Rehabil* 2014, **95**(9):1759-1775.
12. Wade DM, Hankins M, Smyth DA, Rhone EE, Mythen MG, Howell D, Weinman JA: **Detecting acute distress and risk of future psychological morbidity in critically ill patients: Validation of the Intensive care psychological assessment tool**. *Crit Care* 2014, **18**(5):519.
13. Herdman M, Gudex C, Lloyd A, Janssen M, Kind P, Parkin D, Bonsel G, Badia X: **Development and preliminary testing of the new five-level version of EQ-5D (EQ-5D-5L)**. *Qual Life Res* 2011, **20**(10):1727-1736.
14. van Hout B, Janssen MF, Feng YS, Kohlmann T, Busschbach J, Golicki D, Lloyd A, Scalone L, Kind P, Pickard AS: **Interim scoring for the EQ-5D-5L: mapping the EQ-5D-5L to EQ-5D-3L value sets**. *Value Health* 2012, **15**(5):708-715.
15. Zigmond AS, Snaith RP: **The hospital anxiety and depression scale**. *Acta Psychiatr Scand* 1983, **67**(6):361-370.
