# Supplementary material for: Early In-Bed Cycle Ergometry With Critically Ill, Mechanically Ventilated Patients: Statistical Analysis Plan for CYCLE (Critical Care Cycling to Improve Lower Extremity Strength), an International, Multicenter, Randomized Clinical Trial
Source: JMIR Res Protoc. 2024 Oct 28;13:e54451. doi: 10.2196/54451 (PMC11555464; doi:10.2196/54451)
Supplement: Multimedia Appendix 3 [file resprot_v13i1e54451_app3.pdf]

January/Janvier 22, 2018

DC0190GP

Canadian Institutes of Health Research / Instituts de recherche en santé du Canada

Notice of Decision / Avis de décision

Application Number/Numéro de la demande: 390639

Committee Code/Code du comité: RC1

Applicants/Candidats: Dr. Michelle Elisabeth Kho

With/Avec:				
	Docteur P. Archambault	Dr. I. Ball	Dr. S. Berney	Dr. K. Burns
	Dr. D. Cook	Docteur F. D'Aragon	Dr. P. Dodek	Dr. E. Duan
	Dr. A. Fox-Robichaud	Dr. M. Herridge	Dr. T. Karachi	Dr. S. Mehta
	Dr. J. Muscedere	Dr. A. Pastva	Dr. J. Pellizzari	Dr. B. Rochwerg
	Dr. J. Rudkowski	Dr. A. Seely	Dr. J. Tarride	Dr. L. Thabane
	Mr. V. da Silva			

Institution paid/ Établissement payé: McMaster University

Title/Titre: CYCLE: An international, multi-centre, randomized clinical trial of early in-bed cycling for mechanically ventilated patients

Primary Inst./ Inst. principal: Musculoskeletal Health and Arthritis / Appareil locomoteur et arthrite

Other Related Inst./ Autres inst. connexes: Circulatory and Respiratory Health / Santé circulatoire et respiratoire; Aging / Vieillissement

Competition Outcome/Résultats du concours: Project Grant / Subvention Projet

September/Septembre 15, 2017

Number in competition/Nbre de demandes dans le concours: 3415

Number approved/Nbre de demandes approuvées: 512

Decision on your application/ Décision sur votre demande: Approved / Approuvée

Total Funding Amount:/ Montant total du financement: $1,980,584

Term/Durée: 4 **yrs/ans** 0 **months/mois**

Peer Review Committee Recommendation, for your information and use/
Recommandation du comité d'examen par les pairs, pour fins d'information et d'utilisation:

Committee/Comité: Randomized Controlled Trials / Essais contrôlés randomisés

Number reviewed/ Nbre de demandes examinées: 52

Number approved in that committee/ Nbre de demandes approuvées dans ce comité: 7

Application rank within the committee/ Rang de la demande dans ce comité: 2

Percent Rank Within the Committee/ Rang en pourcentage au sein du comité: 98.04%

Rating/ Cote: 4.42

This document is for information only. Official payment is stated on the CIHR Authorization for Funding.
Document à titre d'information seulement. Le paiement officiel est indiqué sur l'autorisation de financement des IRSC.

Canadian Institutes of Health Research
160 Elgin Street, 9th Floor
Address Locator 4809A
Ottawa, Ontario K1A 0W9

Instituts de recherche en santé du Canada
160, rue Elgin, 9e étage
Indice de l'adresse 4809A
Ottawa (Ontario) K1A 0W9

Institute of Aboriginal Peoples' Health
Institute of Aging
Institute of Cancer Research
Institute of Circulatory and Respiratory Health
Institute of Gender and Health
Institute of Genetics
Institute of Health Services and Policy Research
Institute of Human Development and Child and Youth Health
Institute of Infection and Immunity
Institute of Musculoskeletal Health and Arthritis
Institute of Neurosciences, Mental Health and Addiction
Institute of Nutrition, Metabolism and Diabetes
Institute of Population and Public Health

Institut de la santé des Autochtones
Institut du vieillissement
Institut du cancer
Institut de la santé circulatoire et respiratoire
Institut de la santé des femmes et des hommes
Institut de génétique
Institut des services et des politiques de la santé
Institut du développement et de la santé des enfants et des adolescents
Institut des maladies infectieuses et immunitaires
Institut de l'appareil locomoteur et de l'arthrite
Institut des neurosciences, de la santé mentale et des toxicomanies
Institut de la nutrition, du métabolisme et du diabète
Institut de la santé publique et des populations

January 22, 2018

Dr. Michelle Elisabeth Kho
McMaster University
School of Rehabilitation Science
Institute of Applied Health Sciences, Rm 403
1400 Main Street West
Hamilton, Ontario L8S 1C7

Dear Dr. Kho,

On behalf of the Canadian Institutes of Health Research (CIHR), I am pleased to inform you that your application entitled “CYCLE: An international, multi-centre, randomized clinical trial of early in-bed cycling for mechanically ventilated patients”, submitted to the Project Grant – Fall 2017 competition, has been approved for funding. It has been identified as a large grant which CIHR defines as those within the top 2% of the total grant value within the competition.

In the current competition, a total of 69 applications were identified as large grants and were ranked for potential funding from the specific large grant funding envelope of $18,465M.

Your application reviews and competition results can be accessed through ResearchNet. If you are unable to view these documents, please contact us at support@cihr-irsc.gc.ca. Your Authorization for Funding will follow in the mail.

As CIHR does not notify co-applicants of the decision, we ask that you inform those individuals involved, along with their research institutions (if different from your own) of the outcome of this application.

Should you have any questions, please do not hesitate to communicate with a Processing Officer in the Contact Centre at 613-954-1968 or by e-mail: support@cihr-irsc.gc.ca.

Congratulations on your success in this competition.

Sincerely,

Martine Lafrance

Martine Lafrance, Ph.D.

466772-201709PJT-RC1-390639-102217-PJTAL

CIHR IRSC
Discoveries for life / Découvertes pour la vie

Canada

Manager, Project Grant Program
Program Design and Delivery Branch

Canadian Institutes of Health Research
160 Elgin Street, 9th Floor
Address Locator 4809A
Ottawa, Ontario K1A 0W9

Instituts de recherche en santé du Canada
160, rue Elgin, 9e étage
Indice de l'adresse 4809A
Ottawa (Ontario) K1A 0W9

Institute of Aboriginal Peoples' Health
Institute of Aging
Institute of Cancer Research
Institute of Circulatory and Respiratory Health
Institute of Gender and Health
Institute of Genetics
Institute of Health Services and Policy Research
Institute of Human Development and Child and Youth Health
Institute of Infection and Immunity
Institute of Musculoskeletal Health and Arthritis
Institute of Neurosciences, Mental Health and Addiction
Institute of Nutrition, Metabolism and Diabetes
Institute of Population and Public Health

Institut de la santé des Autochtones
Institut du vieillissement
Institut du cancer
Institut de la santé circulatoire et respiratoire
Institut de la santé des femmes et des hommes
Institut de génétique
Institut des services et des politiques de la santé
Institut du développement et de la santé des enfants et des adolescents
Institut des maladies infectieuses et immunitaires
Institut de l'appareil locomoteur et de l'arthrite
Institut des neurosciences, de la santé mentale et des toxicomanies
Institut de la nutrition, du métabolisme et du diabète
Institut de la santé publique et des populations

January 22, 2018

Dr. Michelle Elisabeth Kho
McMaster University
School of Rehabilitation Science
Institute of Applied Health Sciences, Rm 403
1400 Main Street West
Hamilton, Ontario L8S 1C7

Dear Dr. Kho,

Congratulations on your success in the recent Canadian Institutes of Health Research (CIHR) Project Grant - Fall 2017 competition.

Your application was reviewed by your peers and considered to be of exceptionally high quality. You should take great pride in this achievement, particularly given the highly competitive nature of CIHR funding. As you know, peer review is the cornerstone of our research funding system. This process is made possible because of the volunteerism of individuals who generously gave their time to review your application. We are continuously recruiting and retaining the most accomplished innovative and creative scientists to review health research proposals. As a CIHR-funded researcher, you are encouraged to participate should you be invited to serve in the peer review process for future competitions.

To highlight your achievements and to communicate the value of health research to Canadians, we encourage you to work with your institution to promote your research. To support you in this activity, CIHR has developed guidelines on public communication available at: www.cihr-irsc.gc.ca/e/30789.html.

Once again, I offer you my congratulations and best wishes for success in your research.

Yours sincerely,

Roderick R. McInnes

Roderick R. McInnes, CM, OOnt, MD, PhD
Acting President

466455-201709PJT-RC1-390639-102217-CLPJT

**Review Type / Type d'évaluation:**	Reviewer 1 / Évaluateur 1
**Name of Applicant / Nom du chercheur:**	Kho, Michelle
**Application No. / Numéro de demande:**	390639
**Agency / Agence:**	CIHR/IRSC
**Competition / Concours:**	Project Grant/Subvention Projet
**Committee / Comité:**	Randomized Controlled Trials/Essais contrôlés randomisés
**Title / Titre:**	CYCLE: An international, multi-centre, randomized clinical trial of early in-bed cycling for mechanically ventilated patients

Summary of Application/Résumé de la demande:

This is an application for funding for an international, 360-patient concealed open-label RCT in 17 medical-surgical ICUs with blinded outcome assessment at 3 days after ICU discharge. After informed consent, patients will be randomized to receive 30 minutes/day of in-bed cycling (Cycling) in addition to routine physiotherapy interventions, or routine physiotherapy interventions alone (Routine). Current clinical practice guidelines related to PT interventions are adduced from a modest literature base, and recommendations for early rehabilitation in critically ill patients have not been implemented in practice. The data is heterogeneous and arguably equivocal. The role of in-bed cycling is not clear.

The submission is part of the CYCLE Program of Research: Critical care cYCling to improve Lower Extremity strength, which is an interdisciplinary, multi-method, 5-phase research program to investigate the use of early in-bed cycling in critically ill patients who are receiving MV. Extensive pilot work by the applicants demonstrated that protocolized in-bed cycling can start within 3 days of MV, even if patients are deeply sedated. They have undertaken TryCYCLE (NCT01885442, CIHR funded) which prospectively evaluated safety and feasibility in 33 patients, had a high consent rate (92%), and low rate of cycling termination (Phase I) followed by: a) CYCLE Pilot RCT (NCT02377830, CIHR funded) enrolling 66 patients in 7 Canadian centres, with 85% consent, 79% cycling delivery, and 82% blinded outcome ascertainment demonstrating feasibility, b) CYCLE Vanguard (CIHR funded) enrolling 40 patients, added 2 international sites (US, Australia), added a physical function assessment at 3 days after ICU discharge, and refined enrollment and cycling strategies (Phase II). The current submission is the: a) CYCLE RCT and b) CYCLE$ economic evaluation (Phase III). The applicants also have plans for BICYCLE, a behavioural knowledge translation intervention (Phase IV).

Adults admitted to a medical-surgical ICU within the first 4 days of MV and first 7 days of ICU, and who could ambulate independently before hospital admission (with or without a gait aid) are eligible. A web-based, comprehensive and secure randomization service (http://www.randomize.net/) will be utilized. . Stratification by center, medical vs. surgical diagnosis, and age 65 or < 65 years will be undertaken. Patients randomized to the study intervention will receive 30 minutes/ day of in-bed cycling in addition to routine physiotherapy interventions, 5 days per week, during their ICU stay (maximum of 28 days or when able to march on the spot for 2 consecutive days, whichever occurs first). Appropriate exclusion criteria are presented. Participants will be reviewed daily for temporary exemptions precluding cycling. During every cycling session, patients will be carefully monitored for safety. A specialized in-bed cycle ergometer will be used, which provides passive, active- assisted, and active cycling (see above). Cycling will be performed actively or passively as the patient is capable and will be independent of level of consciousness. Routine PT includes, based on the patient's alertness and medical stability, activities to maintain or increase limb range of motion and strength, in- and out- of bed mobility, ambulation, and assistance with optimizing airway clearance and respiratory function.

The primary outcome will be the Physical Function Test for ICU-scored (PFIT-s) measured at 3 days after ICU discharge by assessors blinded to treatment allocation. Patients will be followed throughout their ICU and hospital stay until discharge from the index hospital, death in the index hospital, or transfer to another hospital. To capture the short-term benefits of the intervention, a follow-up call at 60-days after study enrolment will be performed to measure HRQoL and healthcare

resource utilization after discharge for participants alive at hospital discharge (Section 2.8; Other Materials, Questionnaire). A core group of assessors (e.g., physiotherapists or occupational therapists), unaware of the patient's treatment assignment will conduct blinded outcome measures at 3 days after ICU discharge and at hospital discharge. Patients and their family members will be asked not to disclose the patient's assigned treatment to blinded assessors to protect against performance bias. The time frame of 3 days after ICU discharge was chosen by the applicants because it is proximal to the intervention, and some patients may be discharged before 7 days. Also, prior studies documented variable delivery of rehabilitation post-ICU that may contaminate later evaluations.

Secondary outcomes include muscle strength and function, patients' perception of physical function, activities of daily living (ADL) scale, frailty, critical care-related psychological distress, HRQoL, Quality-Adjusted Life Years (QALYs), mortality, hospital discharge location, healthcare utilization (e.g., length of MV, LOS and mortality (ICU, hospital)), and intervention and healthcare costs. An elaborate plan for economic evaluation is planned as well.

The sample size of 360 patients is based on identifying a 1.0 point mean difference between the Cycling and Routine groups for the PFIT-s 3 days after ICU discharge. Psychometric studies of the PFIT identified the minimal clinically important difference was 1.0 points. By logistic regression, analysis of patients enrolled in TryCYCLE and CYCLE pilot studies identified that each 1.0 point increase in PFITs at ICU discharge was associated with a 40% reduction in the composite outcome of death, readmission to ICU, or need for paid assistance after hospital discharge. Based on a standard deviation of 2.5 points at ICU discharge (from CYCLE pilot RCT, no data at 3 days after ICU), a 1.0 point difference between groups and 90% power (0.05 alpha), 266 patients (133 per group) need to be randomized. Based on pilot data, 35% total attrition is anticipated (based primarily on ICU mortality) and so 360 patients overall will be recruited. Forty patients from the CYCLE Vanguard study will contribute to enrollment.

**Review Type / Type d'évaluation:**	Reviewer 1 / Évaluateur 1
**Name of Applicant / Nom du chercheur:**	Kho, Michelle
**Application No. / Numéro de demande:**	390639
**Agency / Agence:**	CIHR/IRSC
**Competition / Concours:**	Project Grant/Subvention Projet
**Committee / Comité:**	Randomized Controlled Trials/Essais contrôlés randomisés
**Title / Titre:**	CYCLE: An international, multi-centre, randomized clinical trial of early in-bed cycling for mechanically ventilated patients

Strengths and Weaknesses/Forces et faiblesses:

Dr. Kho and colleagues submit an application to investigate the role of early exercise via in-bed cycling in patients on mechanical ventilators in ICU in improvement of strength (at 3 days post ICU discharge) and other secondary outcomes including an economic evaluation of the intervention. They hypothesize that the intervention will improve strength and rehabilitation leading to accelerated recovery and decreased long-term disability in ICU survivors. They will examine the effect on quality of life, health care resource utilization and survival up to 60 days post discharge. The subject matter is of immediate relevance and importance in the Canadian health care environment where significantly more ICU utilization and the associated disability that comes with it is anticipated in the future. High quality studies designed to mitigate the deleterious long-term effects of critical illness are aligned with the needs of patients and society as a whole. If the hypothesis is proven, especially with account of cost-effectiveness, the implications should be sweeping not only for the Canadian ICU community but the international community as well.

The approach and methods are very well designed, pre-researched and presented. Successful execution of the pilot and Vanguard phases of study resolve many potential concerns regarding feasibility. This is especially true from the standpoint of obtaining consent where one might envision patients not wanting to be randomized to the standard care group for something as intuitively acceptable as exercise. The applicants made no mention of this in the current submission. Was this a concern for potential enrollees?

The other more potentially relevant issue going forward relates to contamination and blinding. A precise account of unblinding of assessors in the pilot study has been given and considered in the determination of the current target enrollment, but what is the effect of having patients in the same ICU enrolled in both arms of the study? Obviously patients (and their families) cannot be blinded to the therapy they receive but is there a particular concern for patients/families in the control group who witness or become more aware of the nature of the therapeutic intervention? The primary outcome should be relatively objective (reverse placebo type effect notwithstanding) but all of the post discharge assessment relies on self-report. The submission does indicate that patients and families will be asked not to inform their assessors of their randomization allocation. This should be further elaborated to include, for the purposes of the study, with anyone outside of the treating team including other patients and family members, and at a minimum any patient undertaking in-bed cycling should be physically shielded by ICU curtains etc. so that they may not be observed by others. All ICUs may not afford this level of privacy. Given the now established safety profile of in-bed cycling, is there a means of referring to the ‘enhanced exercise program’ without specifically using the term in-bed cycling during the consent process? Would this allow patient blinding where (unless they have previous ICU experience) they could not necessarily identify their standard physiotherapy as the control condition? The applicants indicate that REB approval will be sought from Clinical Trials Ontario for all Ontario sites. This presents an advantage and opportunity for potentially having a focused discussion regarding this with the research ethics body.

The only other major concern relates to the duration and extent of follow-up. The applicants have chosen physical function on day 3 as their primary outcome even though Canadian data has correlated performance on day 7 with predicted physical

function 1 year later. The time frame of 3 days after ICU discharge was chosen by the applicants for practical reasons; because it is proximal to the intervention, and some patients may be discharged before 7 days. Also, prior studies documented variable delivery of rehabilitation post-ICU that may contaminate later evaluations. It appears the final assessment is by interview at 60 days post enrollment. The interview relies on patient self-report on quality of life and account of many details related to health care utilization. Presumably there is no better way via available data systems to track health care utilization and this should be acceptable for the short term. No long-term physical assessment or repeated quality of life, living situation/occupation and other data collection is planned in the submission. Given the excellent quality and tremendous amount of work being undertaken to do this important research it is unclear why the applicants stopped short on inclusion of a 1-year physical, interview and survival assessment which may be very compelling and provide information to absolutely confirm the need for adoption of this intervention in ICUs across Canada and the world. Presumably practicality is the issue or the applicants will garner separate funds to facilitate this inquiry (although this was not described in the phase description of the overall research program). It is strongly advised that the accomplished research team endeavor to collect longer term data.

The submission is a natural progression of a well designed research program which has undertaken extensive pilot work to demonstrate feasibility of the current design. The work to date has been CIHR grant funded and includes a 66 patient RCT pilot followed by a 40 patient vanguard phase. Enrollment was lower than expected in these phases of study, however, the applicants appear to have taken steps to enhance enrollment and the monthly targets for the current submission do not seem to over exaggerate relative to what was seen in the pilot. There is avid support and involvement from the CCCTG and the participating centers all of which are comprised of accomplished and expert critical care trials researchers. There is every reason to expect this trial will be accomplished and provide important findings for consumption by the health care system and Canadians in general.

**Review Type / Type d'évaluation:**	Reviewer 2 / Évaluateur 2
**Name of Applicant / Nom du chercheur:**	Kho, Michelle
**Application No. / Numéro de demande:**	390639
**Agency / Agence:**	CIHR/IRSC
**Competition / Concours:**	Project Grant/Subvention Projet
**Committee / Comité:**	Randomized Controlled Trials/Essais contrôlés randomisés
**Title / Titre:**	CYCLE: An international, multi-centre, randomized clinical trial of early in-bed cycling for mechanically ventilated patients

Summary of Application/Résumé de la demande:

The current proposal is focused on evaluating whether in-bed cycling can improve physical function of critically ill inpatients who are mechanically ventilated. Muscle atrophy and weakness can occur very quickly in mechanically ventilated patients, who are often bed-bound and cannot do traditional physiotherapy. The team proposes that in-bed cycling is more efficient because it requires fewer therapists than traditional mobility exercises, and can be done passively or semi-passively e.g., in the case of sedation. The team proposes a 17 centre randomized controlled trial to compare traditional PT to PT plus in-bed cycling, 5 days weekly.

The PI is an early career investigator in rehab sciences with a CRC and a strong track record in this area. The PI is well supported by her team that includes biostatistics and epidemiology expertise, clinical expertise and a large number of sites committed to the work. The PI has led 3 prior pilot/feasibility studies to inform the current work. Each has led to incremental advances to research in the area, and has informed the design of the current trial.

**Review Type / Type d'évaluation:**	Reviewer 2 / Évaluateur 2
**Name of Applicant / Nom du chercheur:**	Kho, Michelle
**Application No. / Numéro de demande:**	390639
**Agency / Agence:**	CIHR/IRSC
**Competition / Concours:**	Project Grant/Subvention Projet
**Committee / Comité:**	Randomized Controlled Trials/Essais contrôlés randomisés
**Title / Titre:**	CYCLE: An international, multi-centre, randomized clinical trial of early in-bed cycling for mechanically ventilated patients

Strengths and Weaknesses/Forces et faiblesses:

The grant is extremely well-written. It is clear that by building on this idea through several pilots has resulted in a clear and concise direction for the research, and consideration for risks and mitigating strategies.

I think it will be difficult to keep assessors blind to group allocation.

That it has not been evaluated in a multicentre RCT is not sufficient justification. It is clear that enrolment rates for the pilots were relatively low because of pragmatics, which necessitates the large number of centres. The pilots discovered that PT capacity was a limiting factor to enrol participants. That factor has somewhat been addressed with refinements to protocol. Still, 45% were not randomized because of limited PT capacity. Intervention is testing PT plus cycling compared to cycling alone – increased PT time overall. In the real world of financially constrained health care, it will be hard to get more PT time. Perhaps it may not be feasible in the real world if capacity in best case scenario (funded study in keen centres) if half of patients can participate?

Evidence from Cycle pilot and vanguard are not published (except in abstract form). The grant alludes to a few findings from those studies but more clarity is needed since reviewers cannot look up the findings in published trials. For example, the published abstract says average enrolment was 1 patient per site - what was the range? did all sites achieve this or were there sites that did not meet recruitment targets? I would have liked to see some data in the support letters about patient volumes and realistic recruitment targets for each site. Some of the letters had identical sentences about prior experiences with pilot studies - seemed copy and pasted. Nonetheless, the collaborators seem keen to participate and indicate their confidence in contributing to the trial.

**Review Type / Type d'évaluation:**	Reviewer 3 / Évaluateur 3
**Name of Applicant / Nom du chercheur:**	Kho, Michelle
**Application No. / Numéro de demande:**	390639
**Agency / Agence:**	CIHR/IRSC
**Competition / Concours:**	Project Grant/Subvention Projet
**Committee / Comité:**	Randomized Controlled Trials/Essais contrôlés randomisés
**Title / Titre:**	CYCLE: An international, multi-centre, randomized clinical trial of early in-bed cycling for mechanically ventilated patients

Summary of Application/Résumé de la demande:

The proposed trial compares in-bed cycling physiotherapy in addition to routine physiotherapy to routine physiotherapy alone for mechanically ventilated patients at ICUs. The primary research question is whether addition of in-bed cycling improves physical function after ICU discharge. Economic evaluation of the intervention is also proposed. The proposed study design is multicenter two-arm parallel randomized trial with stratified randomization and blinded outcome assessment. Concealment is ensured through the use of web-based randomization service. The primary outcome, Physical Function Test for ICU, is measured once, 3 days after ICU discharge. 360 patients from 17 Canadian and international ICUs are to be recruited overall, accounting for attrition. The proposed primary analysis is t-test or U test comparing the function scores between the two arms. One interim analysis is planned (Haybittle-Peto rule).

**Review Type / Type d'évaluation:**	Reviewer 3 / Évaluateur 3
**Name of Applicant / Nom du chercheur:**	Kho, Michelle
**Application No. / Numéro de demande:**	390639
**Agency / Agence:**	CIHR/IRSC
**Competition / Concours:**	Project Grant/Subvention Projet
**Committee / Comité:**	Randomized Controlled Trials/Essais contrôlés randomisés
**Title / Titre:**	CYCLE: An international, multi-centre, randomized clinical trial of early in-bed cycling for mechanically ventilated patients

Strengths and Weaknesses/Forces et faiblesses:

+ Well-written and well-structured proposal.

+ The trial has potential to directly impact therapy of ICU patients.

+ Extensive CIHR-funded pilot work has been carried out to establish safety and feasibity, recruit and train sites, addressing barriers to enrolment, and provide inputs to sample size calculation.

+ The principal applicant has an impressive track record in leading this research program.

+ Economic evaluation proposed.

- Not clear why a paired t-test is chosen for the main analysis rather than 2-sample t-test.
- Section 2.4 states that the sample size calculation accounts for mortality, which in turn protects from survivor bias. Inflating the sample size to account for loss of power due to attrition does not in itself reduce bias.
- The proposed primary analysis does not adjust for stratification factors.

Review Type/Type d'évaluation: SO Notes /Notes de l'agent scientifique
Name of Applicant/Nom du chercheur: Kho, Michelle Elisabeth
Application No./Numéro de demande: 390639
Agency/Agence: CIHR/IRSC
Competition/Concours: 2017-09-15 Project Grant/Subvention Projet
Committee/Comité: Randomized Controlled Trials/Essais contrôlés randomisés
Title/Titre: CYCLE: An international, multi-centre, randomized clinical trial of early in-bed cycling for mechanically ventilated patients

---

Assessment/Évaluation:

The committee was very enthusiastic about this application and believed its impact may be significant.
This RCT in ICU survivors of critical illness, explores the effect of in-bed cycling plus routine physiotherapy interventions versus routine physiotherapy interventions, with physical function 3 days after discharge from ICU as the primary outcome. An economic evaluation is also planned.

Strengths:

This is an excellent multi-disciplinary team which includes clinicians and researchers with a range of relevant complementary disciplines.
The team has undertaken several CHR-funded preparatory research studies which have contributed to the design of the study, including safety and pilot studies and have included 2 international sites (which addresses feasibility concerns).

Weaknesses/ suggestions:

Limited physiotherapist availability may limit feasibility of the trial. Additional trial centres may be necessary to increase feasibility.
The trial may be subject to problems of lack of blinding.
Duration of follow-up is 60 days – the committee suggested adding a follow-up at 12 months. See reviewer's comments for details.
For specific statistical points – see statistical reviewer's notes.
The committee suggested adding more Canadian sites to address the concern about feasibility.

The description of the economic evaluation lacks sufficient details to allow a complete assessment of its methods.
The applicants plan to administer the EQ-5D on discharge from ICU and at 60 days – at each time point, patients are asked about current quality of life and before admission to ICU.In an unblinded trial, the committee felt that there is a high risk of bias in asking participants to imagine a typical day prior to their ICU admission, and then using this as a primary effectiveness outcome for the economic evaluation. Further support for this approach should be provided in addition to citation #92. Alternatively, and preferably, consideration might be given to making the analysis a cost-effectiveness analysis, using the primary outcome of the clinical trial.

Budget:

Trainee travel was not justified. 2 postdoc trainees are not justified. These costs should be removed from the budget.

**Review Type/Type d'évaluation:**	SO Notes /Notes de l'agent scientifique
**Name of Applicant/Nom du chercheur:**	Kho, Michelle Elisabeth
**Application No./Numéro de demande:**	390639
**Agency/Agence:**	CIHR/IRSC
**Competition/Concours:**	2017-09-15 Project Grant/Subvention Projet
**Committee/Comité:**	Randomized Controlled Trials/Essais contrôlés randomisés
**Title/Titre:**	CYCLE: An international, multi-centre, randomized clinical trial of early in-bed cycling for mechanically ventilated patients

Assessment/Évaluation:
